# Diabetes mellitus and hard braking events in older adult drivers

**DOI:** 10.1186/s40621-024-00508-2

**Published:** 2024-06-05

**Authors:** Difei Liu, Stanford Chihuri, Howard F. Andrews, Marian E. Betz, Carolyn DiGuiseppi, David W. Eby, Linda L. Hill, Vanya Jones, Thelma J. Mielenz, Lisa J. Molnar, David Strogatz, Barbara H. Lang, Guohua Li

**Affiliations:** 1 Department of Epidemiology, Mailman School of Public Health, Columbia University, New York, NY 10032 USA; 2 Department of Anesthesiology, Vagelos College of Physicians and Surgeons, Columbia University, 622 West 168th Street, New York, NY PH5-534, 10032 USA; 3 Department of Psychiatry, Vagelos College of Physicians and Surgeons, Columbia University, New York, NY 10032 USA; 4 Department of Biostatistics, Mailman School of Public Health, Columbia University, New York, NY 10032 USA; 5 Department of Emergency Medicine, University of Colorado School of Medicine, Aurora, CO 80045 USA; 6 VA Eastern Colorado Geriatric Research Education and Clinical Center, Aurora, CO 80045 USA; 7 Department of Epidemiology, Colorado School of Public Health, University of Colorado Anschutz Medical Campus, Aurora, CO 80045 USA; 8 College of Engineering, University of Michigan Transportation Research Institute, Ann Arbor, MI 48109 USA; 9 School of Public Health, University of California San Diego, La Jolla, San Diego, CA 92093 USA; 10 Department of Health, Behavior and Society, Johns Hopkins Bloomberg School of Public Health, Baltimore, MD 21205 USA; 11 Columbia Center for Injury Science and Prevention, Columbia University Irving Medical Center, New York, NY 10032 USA; 12 Bassett Research Institute, Bassett Healthcare Network, Cooperstown, NY 13326 USA

**Keywords:** Aging, Cohort study, Diabetes, Driving safety, Hard braking event, Motor vehicle accident, Older adults

## Abstract

**Background:**

Diabetes mellitus (DM) can impair driving safety due to hypoglycemia, hyperglycemia, diabetic peripheral neuropathy, and diabetic eye diseases. However, few studies have examined the association between DM and driving safety in older adults based on naturalistic driving data.

**Methods:**

Data for this study came from a multisite naturalistic driving study of drivers aged 65–79 years at baseline. Driving data for the study participants were recorded by in-vehicle recording devices for up to 44 months. We used multivariable negative binomial modeling to estimate adjusted incidence rate ratios (aIRRs) and 95% confidence intervals (CIs) of hard braking events (HBEs, defined as maneuvers with deceleration rates ≥ 0.4 g) associated with DM.

**Results:**

Of the 2856 study participants eligible for this analysis, 482 (16.9%) reported having DM at baseline, including 354 (12.4%) insulin non-users and 128 (4.5%) insulin users. The incidence rates of HBEs per 1000 miles were 1.13 for drivers without DM, 1.15 for drivers with DM not using insulin, and 1.77 for drivers with DM using insulin. Compared to drivers without DM, the risk of HBEs was 48% higher for drivers with DM using insulin (aIRR 1.48; 95% CI: 1.43, 1.53).

**Conclusion:**

Older adult drivers with DM using insulin appear to be at increased proneness to vehicular crashes. Driving safety should be taken into consideration in DM care and management.

## Background

Population aging is a global public health issue (World Health Organization 2024). In the United States, older adults (i.e., those aged 65 years and older) accounted for 16% of the population in 2019, and the percentage is expected to increase to 21.6% by 2040 (Administration for Community Living 2021). As a result, the number of older drivers will also increase. By 2050, there will be one older adult in every four licensed US drivers, and the same trend has been observed in Japan, Canada, and the European Union (Savoie et al. 2022; Zhao and Yamamoto 2021). For many older people, driving is an instrumental activity in maintaining mobility, which is necessary for their independence and life quality (Moon and Park 2020). However, impairment in driving performance is common among older adults due to age-related diseases and polypharmacy use (Falkenstein et al. 2020). Furthermore, older adult drivers have a higher rate of fatal crash involvement per mile driven and a higher case fatality rate given a crash than younger adult drivers (Li et al. 2003; Pitta et al. 2021).

Diabetes mellitus (DM) is a chronic metabolic disease characterized by high blood glucose levels. Specifically, type 1 diabetes is caused by insufficient insulin secretion, and type 2 diabetes is caused by resistance of insulin effect and deficiency of compensatory insulin secretion as response (American Diabetes Association 2013). The reported prevalence of DM is 14.7% for US adults and 29.2% for older adults (Centers for Disease Control and Prevention 2023). Previous research on DM and driving safety has identified hypoglycemia as the most eminent risk factor for crashes (Keten 2021). Complications impairing driving safety also include diabetic peripheral neuropathy, hyperglycemia, and diabetic eye disease (Graveling and Frier 2015). In addition, insulin treatment, which is received by about 31% of DM patients, has been recognized as an influential factor of risky driving (Hostiuc et al. 2016; Trief et al. 2016).

Previous studies indicate that DM is associated with a slightly increased crash risk, particularly among drivers with insulin-dependent DM (Hostiuc et al. 2016; Kagan et al. 2010). Most studies examining the relationship between DM and crash risk failed to take exposure to driving into consideration, which may mask the increased risk associated with DM if diabetic drivers reduce driving as a mechanism of self-regulation (Kagan et al. 2010). Moreover, many studies measured driving performance using driving simulators or self-reported data (Blanchard et al. 2010; Zöller et al. 2019). To overcome these limitations, researchers have conducted naturalistic driving studies in recent years and the advantages of accurately measuring driving exposure and outcomes with the naturalistic driving study design have been recognized (Singh and Kathuria 2021). When there are insufficient data on crash events in a naturalistic driving study to evaluate a putative risk factor, using proxy measures, such as hard braking events (HBEs), can contribute to more reliable risk estimation (Guo et al. 2010).

In the present study, we assessed the association between DM and the incidence of HBEs among older adult drivers by using naturalistic driving data from a multisite, prospective cohort study. We hypothesize that the incidence rate of HBEs per mile driven for older adult drivers with DM (in particular for insulin users) is significantly higher than their counterparts without DM, with adjustment for demographic and other characteristics.

## Methods

We used data from the Longitudinal Research on Aging Drivers (LongROAD) project, a naturalistic driving study aimed at understanding factors related to driving safety among older drivers. The LongROAD project enrolled a total of 2990 active drivers aged 65–79 years without significant cognitive impairment. Enrollment and baseline assessment were completed between July 2015 and March 2017 at primary care clinics and healthcare systems in five study sites: Ann Arbor, MI; Baltimore, MD; Cooperstown, NY; Denver, CO; and San Diego, CA. Study participants were followed for up to 44 months through the in-vehicle data recording device “DataLogger” (Danlaw, Inc., Novi, Michigan, USA) installed in their primary vehicles at the time of enrollment. Research protocols for the LongROAD project were reviewed and approved by the institutional review boards of the participating institutions and a certificate of confidentiality for the project was obtained from the National Institutes of Health. Informed consent was obtained from all participants. The overall response rate during the three-year follow-up was 85.3%. The methods and study design of the LongROAD project are described in detail elsewhere (Li et al. 2017). The present analysis included 2856 (95.5%) study participants after excluding 116 with unknown DM status and 18 with missing driving data.

The exposure variable in this study was DM with and without insulin treatment, measured through self-report and medication review. As a part of the baseline assessment, each study participant was asked the following question: Have you ever had, or have you ever been told by a doctor or other health professional, that you have diabetes? Respondents with an affirmative answer were classified as having DM and those with a negative answer as having no DM. Study participants with DM were further divided into two categories: DM without insulin use if not receiving insulin treatment, and DM with insulin use if receiving insulin treatment. Insulin treatment was determined based on medications used by the study participants at baseline. Data on medications and supplements currently used at baseline by each study participant were collected through the “brown-bag review” method (Li et al. 2017). While scheduling the baseline assessment, trained research staff asked the participants to bring all current medications (both over-the-counter and prescribed) and supplements with them for review. A separate data form was used to record information for each medication. For each participant, up to 50 medications and supplements were recorded during the “brown-bag review” (Li et al. 2017).

The outcome variable in this study was HBE, defined as a maneuver with a longitudinal deceleration rate ≥ 0.4 g. HBEs, commonly known as *near-crashes*, are widely used for measuring safety performance in naturalistic driving studies (Guo et al. 2010; Keay et al. 2013; Chevalier et al. 2017; Eby et al. 2019). Data on HBEs and driving exposures were collected through the in-vehicle recording device transmitted to a secure computer server and processed monthly according to predefined parameters (Li et al. 2017). The incidence rate of HBEs per mile driven was used as a surrogate measure of driving safety in this study.

Covariates considered in this study were self-reported demographic and socioeconomic characteristics (e.g., age, gender, race/ethnicity, marital status, education level, annual household income), urbanicity of residence (urban, suburban, and rural), and number of medications currently used at baseline.

Exploratory analysis was conducted to examine the prevalence of DM and the incidence rates of HBEs according to DM status and other characteristics. Multivariable negative binominal modeling was used to estimate adjusted incidence rate ratios (aIRRs) and 95% confidence intervals (95% CIs) of HBEs associated with DM. The multivariable negative binominal models adjusted for demographic and socioeconomic characteristics, urbanicity of residence, and number of medications used. The logarithm of total mileage was included as the offset in models. We chose the multivariable negative binomial model because HBEs were over-dispersed in the study sample. All data analysis was performed using SAS OnDemand for Academics (SAS Institute Inc., Cary, NC, USA).

## Results

### Baseline characteristics of the study sample

Of 2856 drivers studied, 41.6% were 65–69 years of age, 47.1% were male, and 85.5% were non-Hispanic White (Table 1). The majority of the study participants were currently married (62.8%), had a bachelor’s or an advanced degree (63.7%), an annual household income ≥ $50,000 (73.8%), and lived in urban areas (72.3%) (Table 1). On average, the study participants used 8.0 (± 5.1 SD) medications, with 32.8% using 10 or more medications and a median of 7 medications (Table 1).

**Table 1:** Prevalence of diabetes mellitus (DM) with and without insulin use by driver characteristics, the Longitudinal Research on Aging Drivers (LongROAD) Study

Variable	Total *n* ^a^	DM without Insulin Use *n* (%)	DM with Insulin Use *n* (%)
Age (years)			
65–69	1,187	149 (12.6)	67 (5.6)
70–74	989	118 (11.9)	31 (3.1)
75–79	680	87 (12.8)	30 (4.4)
Gender			
Male	1,345	190 (14.1)	78 (5.8)
Female	1,511	164 (10.9)	50 (3.3)
Race/Ethnicity			
White, non-Hispanic	2,442	267 (10.9)	92 (3.8)
Black, non-Hispanic	212	49 (23.1)	24 (11.3)
Other	198	37 (18.7)	12 (6.1)
Marital Status			
Married	1,793	220 (12.3)	78 (4.4)
Non-married	1,036	131 (12.6)	50 (4.8)
Education Level			
High school or lower	324	74 (22.8)	25 (7.7)
Between high school and bachelor	702	102 (14.5)	43 (6.1)
Bachelor’s degree	664	66 (9.9)	23 (3.5)
Advanced degree	1,157	111 (9.6)	37 (3.2)
Annual Household Income			
<$50,000	749	113 (15.1)	50 (6.7)
$50,000-$79,999	696	97 (13.9)	30 (4.3)
$80,000-$99,999	406	47 (11.6)	14 (3.5)
≥$100,000	900	85 (9.4)	29 (3.2)
Urbanicity			
Urban	2,066	257 (12.4)	91 (4.4)
Suburban/rural	790	97 (12.3)	37 (4.7)
Number of Medications Used			
0–4	724	33 (4.6)	4 (0.6)
5–9	1,112	150 (13.5)	22 (2.0)
≥10	895	162 (18.1)	102 (11.4)

^a^Total number within variables may vary due to missing data

### Prevalence of DM

Overall, 482 (16.9%) of the study participants reported having DM at baseline; of them, 354 (12.4%) were insulin non-users, and 128 (4.5%) were insulin users. The prevalence of DM was significantly higher among drivers who were 65–69 years of age (18.2%), male (19.9%), or non-Hispanic Black (34.4%) (Table 1). The prevalence of DM decreased with increased education level and annual household income (Table 1). There was a strong positive correlation between the prevalence of DM and the number of medications used (Table 1). The overall prevalence of DM was not significantly associated with marital status and urbanicity of residence. In general, distributions of DM without insulin use and DM with insulin use followed the same patterns across demographic and other variables (Table 1).

### Incidence of HBEs

**Fig. 1:**
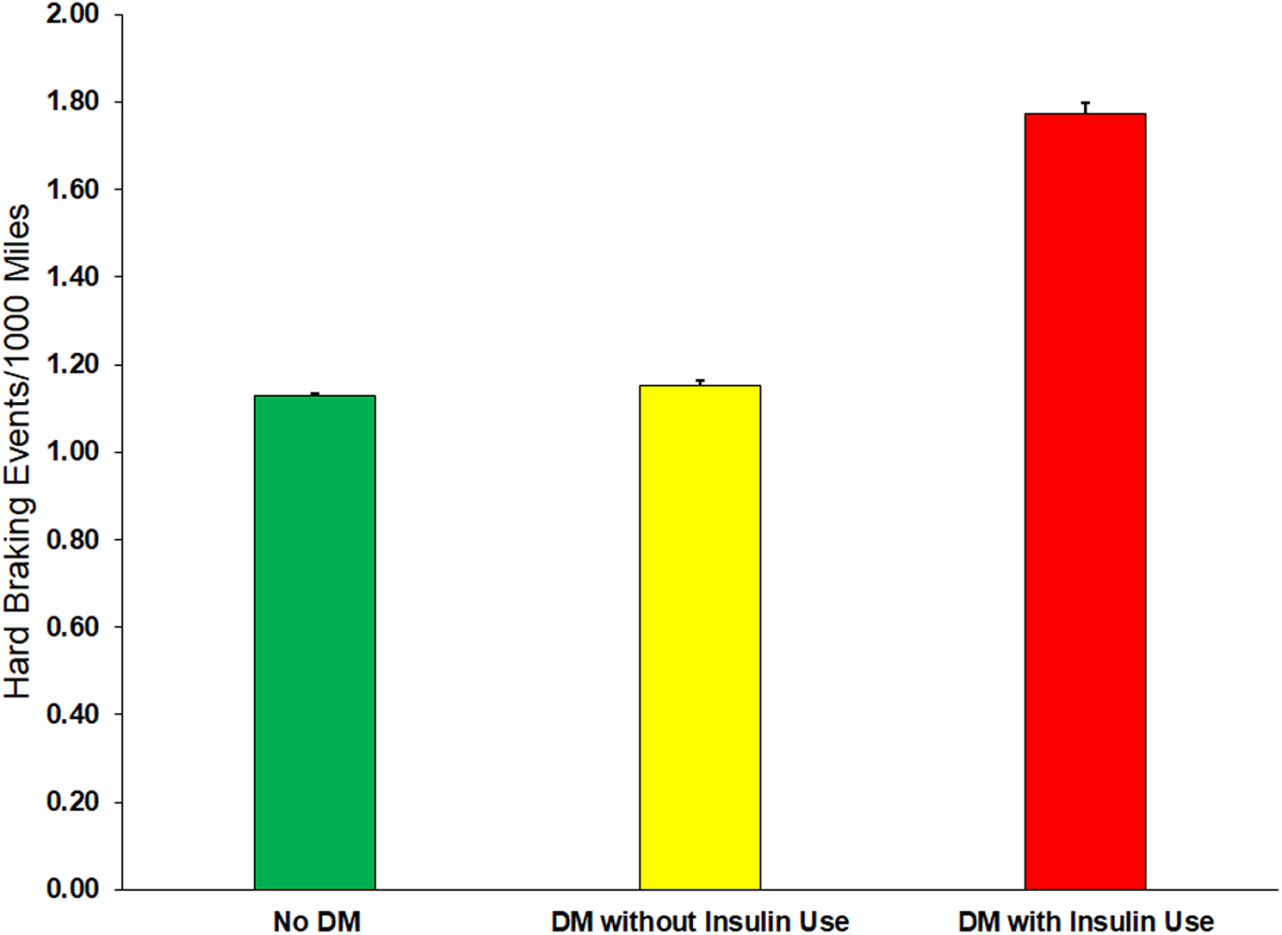
Incidence rates and standard errors of hard braking events per 1,000 miles driven according to DM and insulin treatment, the Longitudinal Research on Aging Drivers (LongROAD) Study

During the follow-up, the in-vehicle data recording devices captured driving data for a total of 64,297,858 miles and 74,558 HBEs, yielding an overall incidence rate of 1.16 HBEs per 1000 miles. The incidence rates of HBEs were 1.13 (95% CI: 1.12, 1.14) for drivers without DM, 1.15 (95% CI: 1.13, 1.18) for drivers with DM not using insulin, and 1.77 (95% CI: 1.72, 1.82) for drivers with DM using insulin (Fig. 1). For those with DM as a whole, the incidence rate of HBEs was 1.32 (95% CI: 1.30, 1.34). Significantly higher incidence rates of HBEs were also found in drivers who were older, female, non-Hispanic White, not currently married, had lower education, lived in urban areas, or used 10 or more medications (Table 2).

**Table 2:** Incidence rates (IRs) of hard braking events per 1,000 miles driven, adjusted incidence rate ratios (aIRRs) and 95% of confidence intervals (CIs) by driver characteristics, the Longitudinal Research on Aging Drivers (LongROAD) Study

Variable	Total miles	Number of HBEs	IR (95% CI)	aIRR (95% CI)
Diabetes Mellitus and insulin treatment				
No DM	53,628,020	60,464	1.13 (1.12, 1.14)	1.00
DM without Insulin Use	7,766,936	8,948	1.15 (1.13, 1.18)	0.97 (0.95, 0.99)
DM with Insulin Use	2,902,902	5,146	1.77 (1.72, 1.82)	1.48 (1.43, 1.53)
Age (years)				
65–69	28,583,660	31,317	1.10 (1.08, 1.11)	1.00
70–74	22,504,328	26,269	1.17 (1.15, 1.18)	1.10 (1.08, 1.12)
75–79	13,209,870	16,972	1.28 (1.27, 1.30)	1.21 (1.19, 1.24)
Gender				
Male	32,885,354	37,527	1.14 (1.13, 1.15)	1.00
Female	31,412,503	37,031	1.18 (1.17, 1.19)	1.03 (1.01, 1.04)
Race/Ethnicity				
White, non-Hispanic	55,796,527	61,865	1.11 (1.10, 1.12)	1.00
Black, non-Hispanic	4,562,533	6,004	1.32 (1.28, 1.35)	0.90 (0.88, 0.93)
Other	3,867,347	6,534	1.69 (1.65, 1.73)	1.27 (1.24,1.31)
Marital Status				
Married	42,451,922	45,850	1.08 (1.07, 1.09)	1.00
Non-married	21,308,045	27,938	1.31 (1.30, 1.33)	1.17 (1.15, 1.19)
Education Level				
High school or lower	6,512,537	7,031	1.08 (1.05, 1.10)	1.00
Between high school and bachelor	16,141,721	20,149	1.25 (1.23, 1.27)	1.13 (1.09, 1.16)
Bachelor’s degree	14,748,052	17,416	1.18 (1.16, 1.20)	1.05 (1.02, 1.09)
Advanced degree	26,687,426	29,773	1.12 (1.10, 1.13)	0.95 (0.92, 0.98)
Annual Household Income				
<$50,000	15,052,356	19,427	1.29 (1.27, 1.31)	1.00
$50,000-$79,999	16,176,146	16,098	1.00 (0.98, 1.01)	0.80 (0.79, 0.82)
$80,000-$99,999	10,351,241	11,400	1.10 (1.08, 1.12)	0.94 (0.91, 0.96)
≥$100,000	20,557,098	25,244	1.23 (1.21, 1.24)	1.02 (0.99,1.04)
Urbanicity				
Urban	42,500,372	59,450	1.40 (1.39, 1.41)	1.00
Suburb/Rural	21,797,486	15,108	0.69 (0.68, 0.70)	0.49 (0.48, 0.50)
Number of Medications Used				
0–4	17,461,042	18,826	1.08 (1.06, 1.09)	1.00
5–9	25,456,741	28,097	1.10 (1.09, 1.12)	0.99 (0.97, 1.01)
≥10	18,689,584	24,723	1.32 (1.31, 1.34)	1.10 (1.08, 1.13)

### Multivariable modeling

With adjustment for age, gender, race/ethnicity, marital status, education level, annual household income, urbanicity, and number of medications used, DM without insulin use was associated with a slightly decreased risk of HBEs (aIRR: 0.97, 95% CI: 0.95, 0.99) and DM with insulin use was associated with a 48% increased risk of HBEs (aIRR: 1.48, 95% CI: 1.43, 1.53) (Table 2). Overall, DM was associated with a 10% increased risk of HBEs (aIRR 1.10; 95% CI: 1.08, 1.12).

## Discussion

Our results indicate that older adult drivers with DM are at a modestly increased risk of HBEs compared to their counterparts without DM and that the increased risk is limited to those using insulin. The increased risk of HBEs associated with insulin users remains significant with adjustment for demographic and other characteristics. Our findings are consistent with previous studies, which reported an increased risk of vehicular crashes among diabetic patients who were using insulin but not among those who were not using insulin (Avalos et al. 2012; Hemmelgarn et al. 2006; Hostiuc et al. 2016; Orriols et al. 2014; Skurtveit et al. 2009).

Our study adds valuable evidence to the existing research literature on DM and driving safety. It has been noted that previous studies controlling for mileage were more likely to detect an increased crash risk associated with DM, as the decrease in mileage of diabetic drivers could bias the assessment of crash risk toward underestimation (Kagan et al. 2010; Maxwell et al. 2023). The results of our study are especially salient for older drivers with DM who are using insulin. Compared with younger diabetic patients, older patients are at an increased risk of hypoglycemia (Deshmukh et al. 2021; Matyka et al. 1997; Sinclair et al. 2015), which may help explain, to some extent, the excess risk of HBEs associated with DM with insulin use found in the present study.

This study has several notable strengths, including the large sample size, naturalistic driving data, and adjustment for demographic characteristics and polypharmacy use. Nevertheless, our results should be interpreted in light of the study limitations. First, the exposure of our study, DM status, was based on self-reported data at baseline. Therefore, it is susceptible to misclassification due particularly to underreporting of DM in those who are not taking insulin and in those who developed incident DM during the follow-up. It is noteworthy that the prevalence of DM reported in our study (16.9%) is much lower than in the US older adult population (29.2%) (Centers for Disease Control and Prevention 2023). This difference is due in part to the inclusion and exclusion criteria of the LongROAD project aimed at recruiting community-dwelling healthy participants who were active drivers with intact cognitive function (Li et al. 2017). Second, the outcomes of our study, HBEs, were surrogates of vehicular crashes, rather than actual crashes. Although HBEs have been found to be correlated with driving ability and crash risk among older adults (Keay et al. 2013; Chevalier et al. 2017; Eby et al. 2019; Liu et al. 2023), the validity of our findings needs to be confirmed through police-reported crash records and objectively collected driving data. Finally, our study sample was not nationally representative. Rather, study participants in the LongROAD project were active drivers who were disproportionately non-Hispanic White with greater education attainment and higher income than the general US older adult population (Li et al. 2017). Therefore, our findings may not be directly generalizable to the US general older adult driver population.

The relationship between DM and driving safety among older adults warrants further investigation given the aging driver population, the high prevalence of DM in older adults, the importance of driving for older adults (Strogatz et al. 2020), and the excess mortality from motor vehicle crashes among older adult drivers (Li et al. 2003; Pitta et al. 2021). In addition to the age-related increased risk of fatal crash involvement, DM itself can accelerate the development of frailty (Aguayo et al. 2019) and thus make older adult drivers with DM more susceptible to crash involvement (Crowe et al. 2020). Therefore, improving driving safety should be integrated into DM care and management programs for older adults. Future research could shed light on the pathways linking DM to excess crash risk by using detailed data on disease severity, treatment, complications such as diabetic peripheral neuropathy, and comorbidities. Clinical management based on individualized risk evaluation, including risk related to driving, could help improve DM care and patient outcomes (American Diabetes Association et al. 2014).

## Conclusion

Older adult drivers with DM who are using insulin are 48% more prone to vehicular crashes than their counterparts without DM or with DM who are not using insulin. Further research on DM and driving safety is warranted to understand better the mechanisms underlying the relationship between DM with insulin use and excess crash risk among older adult drivers. Interventions to ensure driving safety among older adult drivers with DM should be incorporated into diabetes care and management programs.

## Data Availability

Restrictions apply to the availability of these data. Data are available from the author with permission from the AAA Foundation for Traffic Safety and upon execution of a data use agreement.

## Abbreviations

95% CI: 95% Confidence interval
aIRR: Adjusted incidence rate ratio
DM: Diabetes mellitus
HBE: Hard braking event
IR: Incidence rate
LongROAD: Longitudinal Research on Aging Drivers
